# Role of crosstalk between endothelial cells and smooth muscle cells in vascular calcification in chronic kidney disease

**DOI:** 10.1111/cpr.12980

**Published:** 2021-01-27

**Authors:** Yu‐Xia Zhang, Ri‐Ning Tang, Li‐Ting Wang, Bi‐Cheng Liu

**Affiliations:** ^1^ Institute of Nephrology Zhongda Hospital School of Medicine Southeast University Nanjing China; ^2^ Institute of Nephrology Zhongda Hospital Nanjing Lishui People' Hospital Nanjing China

**Keywords:** CKD, crosstalk, endothelial cells, smooth muscle cells, vascular calcification

## Abstract

Chronic kidney disease (CKD) is a severe health problem worldwide, and vascular calcification (VC) contributes substantially to the cardiovascular morbidity and high mortality of CKD. CKD is often accompanied by a variety of pathophysiological states, such as inflammation, oxidative stress, hyperglycaemia, hyperparathyroidism and haemodynamic derangement, that can cause injuries to smooth muscle cells (SMCs) and endothelial cells (ECs) to promote VC. Similar to SMCs, whose role has been widely explored in VC, ECs may contribute to VC via osteochondral transdifferentiation, apoptosis, etc. In addition, given their location in the innermost layer of the blood vessel lumen and preferential reception of various pro‐calcification stimuli, ECs can pass messages to vascular wall cells and communicate with them. Crosstalk between ECs and SMCs via cytokines through a paracrine mechanism, extracellular vesicles, miRNAs and myoendothelial gap junctions also plays a role in VC. In this review, we emphasize the role of intercellular crosstalk between ECs and SMCs in VC associated with CKD.

## Graphical abstract

The crosstalk between vascular endothelial cells and vascular smooth muscle cells plays an important role in the occurrence and progression of CKD‐associated vascular calcification. Endothelial cells and smooth muscle cells interact with each other through inflammation, muscular endothelial gap junction (MEGJ), vasodilator (eg NO), vasoconstrictor (eg ET‐1), pro‐calcification factor (eg BMP), matrix metalloproteinases (MMPs), extracellular vesicles (EVs) and pro‐angiogenic and pro‐fibrotic growth factors (eg VEGF), etc.

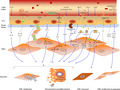

## 1 INTRODUCTION

Chronic kidney disease (CKD) is an extremely serious health problem that causes poor health outcomes and substantial social and economic burdens worldwide. The high cardiovascular morbidity and mortality in the CKD population are mainly caused by extensive and progressive vascular calcification (VC).^1^, ^2^ Notably, in CKD patients, severe intimal calcification (known as atherosclerotic calcification and thought to originate from atherosclerosis) and medial calcification (known as Mönckeberg's sclerosis) are both prominent and can occur simultaneously, and both are accelerated with decreasing kidney function.^2^, ^3^ However, there are currently no effective treatment measures for VC. Although progress has been made in understanding the pathogenesis of VC, it is still largely assumptive and needs to be clarified.

VC was previously regarded as a passive, degenerative disease. With further in‐depth research, calcification has been found to be an active and adjustable process, analogous to bone formation, in which many cells participate, such as endothelial cells (ECs), smooth muscle cells (SMCs) and inflammatory cells.^2^, ^4^ It is generally accepted that vascular smooth muscle cells (VSMCs), the most abundant cell type in the arterial vessel wall, are the ‘main force’ in VC. VSMCs contribute to VC though osteochondrogenic transdifferentiation, extracellular vesicle (EV) release, apoptosis, cellular senescence and so on.^5^ ECs, like SMCs, are one of the major cell types that make up the vessel wall. Many studies have gradually found that ECs also have the potential to undergo osteochondrogenic differentiation to promote VC. In addition, various cell activities are not carried out independently, regardless of the type of calcification. Under various stimulating factors, these cells interact with each other through complex signalling pathways in the vascular microenvironment. VC involves a complex network of multicellular communication.^6^ Given that ECs and SMCs are adjacent wall cells, what kind of crosstalk exists between them in the process of VC? How do circulating pro‐calcification factors affect SMCs that are not in direct contact with the factors? ECs are the innermost layer of the blood vessel wall, meaning that they are the ‘vanguard’ that recognizes a variety of stimulating factors and the ‘liaison’ between pro‐calcification factors and vascular wall cells. Indeed, a series of studies confirmed that crosstalk between ECs and SMCs promotes VC.

The objective of this review is to investigate the role of ECs and the role of crosstalk between ECs and VSMCs in VC.

## 2 VC IN CKD

CKD patients exhibit not only traditional VC risk factors such as older age, diabetes, dyslipidaemia and P but also unique and prominent non‐traditional risk factors and pathological states, including uraemic toxins, hyperphosphataemia, hypercalcaemia, abnormal mineral metabolism, hyperparathyroidism, inflammation, oxidative stress, premature senescence and malnutrition.^7^ Strategies specifically targeting traditional factors do not seem to be effective in CKD, emphasizing the importance of non‐traditional factors as targets for future interventions. With all these risk factors, CKD patients are prone to various types of ectopic calcification. Although medial calcification seems to be the principal type of VC in 7CKD according to previous studies, both intimal calcification and medial calcification are prominent in CKD.^8^, ^9^, ^10^ Although their evoked factors and underlying mechanisms are different, both forms of calcification are accelerated by pro‐calcification factors in various pathological states in the context of CKD and cause serious consequences.

To our knowledge, the current mechanism of VC in CKD involves the following processes: (i) Severe mineral imbalance leads to passive deposition of overloaded calcium and phosphate metabolites such as hydroxyapatite. (ii) Cell death occurs through apoptosis and autophagy, in which apoptotic bodies from SMCs, ECs, macrophages, etc. and necrotic debris may serve as apatite nucleation sites. (iii) Circulating calciprotein particles (CPPs), that is, nucleation complexes of calcium phosphate crystals and chaperone‐binding proteins such as fetuin‐A and matrix Gla protein (MGP), are transformed from primary amorphous CPPs into secondary crystalline CPPs to induce VC in the context of abnormal mineral metabolism in CKD. Additionally, matrix vesicles from bone remodelling are released into circulation and serve as apatite nucleation sites. (iv) Disproportionate promoters (eg calcium (Ca) and phosphate (Pi)) and inhibitors (eg fetuin‐A, osteopontin (OPN), pyrophosphate (PPi) and MGP) of calcification are present. (v) Osteochondral transdifferentiation is induced in SMCs, ECs, stem cells, etc., which may subsequently release extracellular vesicles (EVs) to participate in and regulate calcification. (vi) Elastin is degraded, and the mineralized matrix is re‐established.^11^, ^12^, ^13^, ^14^ CKD, with the pathological conditions of hypercalcaemia, hyperphosphataemia, hyperthyroidism, oxidative stress, inflammation, α‐Klotho deficiency and the accumulation of uraemic toxins, such as indoxyl sulphate (IS) and advanced‐glycation end products (AGEs), will amplify these effects.

## 3 THE ROLE OF SMCS IN CKD‐RELATED VC

It is believed that SMCs play a key role in VC regardless of whether the calcification is medial or intimal, and SMCs have been extensively studied in this regard.^13^ Given their high abundance in the vasculature and their exposure to various pro‐calcification factors, SMCs promote VC through apoptosis, phenotypic transformation (especially osteochondral transdifferentiation), EV release, migration and senescence. Many in vivo and in vitro experiments have confirmed that the above behaviours can be affected by uraemic toxins, including phosphate (Pi), IS, AGEs and pro‐inflammatory cytokines.^15^ In addition, hyperglycaemia, hyperthyroidism, mechanical stress and oxidative stress, which are known as the hallmarks of CKD, are potent inducers of VSMC dysfunction.

It has been shown in vitro and ex vitro that in the CKD milieu, various uraemic toxins, especially calcium and phosphorus, and pathological states induce SMC death. The apoptotic bodies of SMCs and necrotic debris of dead cells are known to act as nucleating foci for calcified crystals.^16^, ^17^ In addition, SMCs have plastic potential and can transdifferentiate from a normal ‘contractile’ phenotype to an osteochondral phenotype by downregulating the expression of SMC markers such as SM22α and SMα‐actin and upregulating the expression of osteochondrogenic markers, such as runt‑related transcription factor 2 (Runx2), osteocalcin (OC), alkaline phosphatase (ALP), bone morphogenetic protein (BMP) and OPN. This phenomenon is linked to the secretion of matrix metalloproteinases (MMP)‐2/9, which can induce the degradation of elastin to re‐establish the mineral matrix.^15^ Notably, in vitro and ex vitro experiments have confirmed that uraemic toxins can induce SMCs to undergo osteogenic transdifferentiation.^10^ The subsequent release of VSMC‐derived vesicles with hydroxyapatite nanocrystals may also produce nucleation sites.^17^ Regarding the plasticity of SMCs, these cells may be able to transdifferentiate into a macrophage‐like phenotype.^13^ VSMC migration and macrophage infiltration, which are believed to play pivotal roles in the onset and development of atherosclerotic lesions, thus further contribute to intimal calcification. In particular, CKD mouse aortic VSMCs exhibited significantly higher migration than normal control VSMCs in ex vivo cell migration assays.^18^ Premature senescence is a characteristic state of CKD, since there are multiple stresses in CKD. Elevated interleukin (IL)‐1β expression coincides with p21 (a senescence‐related protein) expression, BMP2 expression and VC in distal radial arteries in end‐stage renal disease patients. In vitro experiments have confirmed that VSMC senescence induced by IL‐1β may be involved in VC via subsequent osteogenic transdifferentiation. Blocking the senescence process can also attenuate osteogenic transformation.^19^ Overall, previous studies have provided an in‐depth understanding. Therefore, the role of SMCs in VC is not our focus in this review and will not be expounded on in more detail.

## 4 THE ROLE OF ECS IN CKD‐RELATED VC

As one of the main vascular wall cells, ECs are speculated to play an important role in VC, and importance has been attached to them, since they are located in the innermost layer of the lumen and are easily injured when exposed to various pro‐calcification factors. ECs used to be seen as bystanders and victims, ending with endothelial dysfunction, but now, things do not seem to be that simple. The expression of ossification‐related genes in ECs was previously confirmed to change significantly under atherogenic and pro‐inflammatory stimuli in vitro.^20^ In vivo, when the expression of tissue non‐specific alkaline phosphatase (TNAP), an enzyme that regulates mineralization, in ECs is increased, transgenic Tie2‐Cre mice (encoding human TNAP genes) develop extensive VC, which is similar to the result when TNAP is overexpressed in SMCs.^21^ A series of subsequent studies showed that ECs can also undergo a phenotypic switch and act as a source of bone progenitor cells to promote calcification.^22^, ^23^ Compared with the evidence found by tracking the fate of ECs under stimulation by a variety of pro‐calcification factors, stronger evidence has suggested the endothelial origin of chondrocytes and osteoblasts. Chondrocytes and osteoblasts express endothelial markers in calcified lesions in fibrodysplasia ossificans progressiva (FOP), a disease that manifests as ectopic calcification,^23^ and the sites of fractures.^24^ Altogether, these studies demonstrate the critical and active role of ECs in VC.

Notably, endothelial–mesenchymal transition (EndMT) seems to provide a credible explanation for the phenotypic switch and osteogenic potential of ECs.^25^, ^26^ Recognized as the process by which endothelial‐lineage cells lose cell polarity, acquire migratory and invasive characteristics, and differentiate into mesenchymal stem cells (MSCs), EndMT makes ECs plastic with the potential for osteogenic differentiation under different appropriate physiological states. CKD is well known to be characterized by numerous appropriate pathological states, such as inflammation, oxidative stress, hyperglycaemia, hyperparathyroidism and haemodynamic derangement, that can trigger ECs. In addition, ECs may also participate in calcification through apoptosis, communication with SMCs and other mechanisms in the CKD milieu. Nonetheless, the signalling pathways underlying ECs responding to different pathophysiological states during VC are unclear, and related studies are lacking. The following subsections summarize the roles of ECs in VC in the diverse pathological states of CKD.

### 4.1 The role of ECs mediated by inflammation and oxidative stress in CKD‐related VC

CKD is a condition that is characterized by inflammation and oxidative stress. These features are considered to be necessary antecedents regardless of whether physiological or pathophysiological ossification occurs. In CKD, they continue to act on the vasculature and cause damage, such as VC. In vitro, tumour necrosis factor α (TNF‐α) and oxidized low‐density lipoprotein (ox‐LDL) can change the expression of genes linked to ossification in human coronary artery ECs (HCAECs), such as BMP2, MGP and Runx2/core binding factor alpha 1 (Cbfα1).^20^ It was further found that in high‐fat diet‐fed ApoE^−/−^ mice, EndMT could contribute to atherosclerotic lesion calcification.^27^ Inflammation‐ and oxidative stress‐related atherosclerotic stimuli can affect the endothelium and make ECs transdifferentiate into osteoblast‐like cells though EndMT to promote calcification. Sánchez‐Duffhues et al^28^ confirmed in vitro that the pro‐inflammatory cytokines IL‐1β and TNF‐α downregulated BMPR2 (BMP type II receptor) expression in ECs and that the loss of BMPR2 could enhance the osteogenic differentiation of ECs induced by BMP‐9. After screening, the JNK signalling pathway rather than canonical Smad signalling transduction was found to be involved. The BMPR‐JNK signalling axis is a key pathway that regulates inflammation‐induced EndMT and eventually promotes EC osteogenic transdifferentiation. Ox‐LDL was further confirmed to be able to promote the osteogenic activities of ECs along with its pro‐inflammatory and pro‐oxidative effects. Ox‐LDL, which activates the ROS system, can induce the expression of osteochondrogenic transcription factors (Runx2, OPN and Msx2) in ECs in a manner dependent on the production of H_2_O_2_. Additionally, H_2_O_2_ itself is adequate to upregulate the expression of these genes.^29^ These results suggest that ECs affected by inflammation and oxidative stress, hallmarks of CKD, can promote VC.

### 4.2 The role of ECs mediated by high calcium and phosphate levels in CKD‐related VC

The manifestation of Ca and Pi metabolic dysregulation is quite universal among CKD patients. Indeed, high levels of serum Ca, Pi and Ca × Pi products are major risk factors for VC. On the one hand, they are the major components of hydroxyapatite, and thus, they may contribute directly to nanocrystal deposition in the vasculature as their concentrations increase.^30^ On the other hand, they can induce osteogenic/chondrogenic differentiation, apoptosis, EV release and so on in VSMCs to promote VC,^30^ and Pi is considered the main driver in these processes.^31^ Similar to its effects on SMCs, Pi can also cause apoptosis and phenotypic transdifferentiation in ECs.^32^, ^33^, ^34^ It has been confirmed in vitro that the ERK1/2/microRNA‐21 pathway mediates high Pi‐induced EC apoptosis.^32^ Inhibition of the ERK/MAPK pathway can protect against EC apoptosis by upregulating BMP4 expression in ECs exposed to Pi.^33^ Under stimulation by Pi, ECs can change not only their morphology but also the expression of cell markers, a necessary process in EndMT, which manifests as decreases in the expression of endothelial markers (VE‐cadherin) and increases in the expression of the interstitial markers (S100A4) and master transcription factors of EndMT (SNAIL, SLUG and TWIST).^34^ EndMT enables ECs to acquire osteochondrogenic potential, as noted. Ultimately, apoptosis and EndMT in ECs mediated by Pi may further promote VC in CKD.

### 4.3 The role of ECs mediated by high‐glucose levels in CKD‐related VC

Diabetic nephropathy is the main cause of CKD, and CKD patients with hyperglycaemia are relatively prone to VC. Yao et al^25^ verified the contribution of ECs to VC in diabetic mice through EndMT using a cell tracing technique. Our team has also previously demonstrated that in vitro high‐glucose levels can transform human aortic ECs (HAECs) into MSCs through EndMT, which then differentiate into chondrocytes and osteoblasts. Activation of the Snail signalling pathway may partly mediate this process,^35^ and inflammation may also be involved in by IL‑β activation via the PKCβ pathway.^36^ In addition, apoptosis is often related to calcification as a prelude to calcium deposition, and endothelial apoptosis mediated by high‐glucose levels increases.^37^ At the same time, high‐glucose levels lead to an imbalance in the mineral metabolism of ECs by increasing calcium and potassium uptake and can lead to abnormal endothelial nitric oxide synthase (eNOS) phosphorylation and OPN overexpression through the p38 pathway, which eventually disrupts endothelial function and leads to VC.^37^ These data suggest that ECs can promote VC through EndMT osteogenic transdifferentiation, inflammation, apoptosis, unbalanced mineral metabolism and dysfunction in the context of stimulation by high‐glucose concentrations and that many signalling pathways are involved.

### 4.4 The role of ECs mediated by high parathyroid hormone (PTH) levels in CKD‐related VC

Secondary hyperparathyroidism characterized by an elevated PTH level is very prominent in CKD patients. PTH, as a significant bridge, is involved in bone metabolism and cardiovascular disease.^38^ Studies have reported that PTH may directly cause dysfunction in the endothelium and lead to VC. However, the underlying mechanism remains unclear. Cheng et al^39^ found in vitro that PTH promotes osteoblast transdifferentiation in ECs via the (Erk)1/2 and NF‑κB signalling pathways. PTH stimulation may directly upregulate the expression of osteogenic markers, such as BMP2, BMP4, Runx2 and ALP. Our team previously found that PTH induced the expression of MSC markers in ECs in vivo and in vitro, that is, PTH induced EndMT. Then, these cells further differentiated into chondrocyte‐like cells and participated in the formation of CKD aortic calcification, in which enhancement of the nuclear localization of β‐catenin was involved.^40^ Cinacalcet, a calcimimetic that decreases PTH levels without increasing the levels of circulating calcium and phosphorus, can reduce aortic calcification in uraemic rats by blocking EndMT‐mediated osteogenic transdifferentiation.^41^ In conclusion, ECs can promote VC through osteogenic transdifferentiation during exposure to PTH in CKD. In addition, PTH can also induce the expression of voltage‐sensitive calcium channels in ECs,^42^ which may also be involved in calcification.

### 4.5 The role of ECs mediated by mechanical stress in CKD‐related VC

The existence of pathophysiological states such as hypertension and hyperlipidaemia in CKD and dialysis treatments means that patients often have blood flow disorders, which increases mechanical stimulation to the vascular wall. Mechanical stress is believed to be essential in physiological ossification of the bone and perhaps also in pathological ossification. It can also induce the differentiation and maturation of preosteoblasts into osteoblasts and the redirection of other progenitor cells into osteoblast‐like cells.^43^, ^44^ In addition to cytokine stimulation, mechanical stress exposure may also lead to ECs contributing to the initiation of VC. Mechanical stimuli can be transduced into altered gene expression or converted into biochemical signals in ECs. In vitro, mechanical stress causes ECs to upregulate the expression of pro‐osteogenic factors (BMP‐2 and Sprouty‐1), and interestingly, it can further influence the expression of osteogenic genes in vascular fibroblasts and VSMCs when they are co‐cultured with ECs stimulated with mechanical stress or medium from stimulated ECs; however, the underlying crosstalk mechanism is unknown.^45^ This suggests a potential essential role for ECs in initiating VC. Shear stress (a mechanical force) has corresponding roles in inducing endothelial transdifferentiation. In a mitral valve calcification model, ECs localized along the mechanically stretched valve expressed osteoblast markers such as OC, indicating ongoing transdifferentiation.^46^ In addition, a series of studies showed that mechanical stress could promote atherosclerosis, which often eventually leads to calcification through EndMT.^27^, ^47^ Evidence shows that low blood shear stress contributes to the formation of atherosclerotic calcified plaques and that the endothelium and its production of nitric oxide (NO) are crucial for the calcification process.^48^ What is certain is that ECs actively respond to mechanical stimuli to regulate VC.

## 5 CROSSTALK BETWEEN ECS AND SMCS IN CKD‐RELATED VC

**FIGURE 1:**
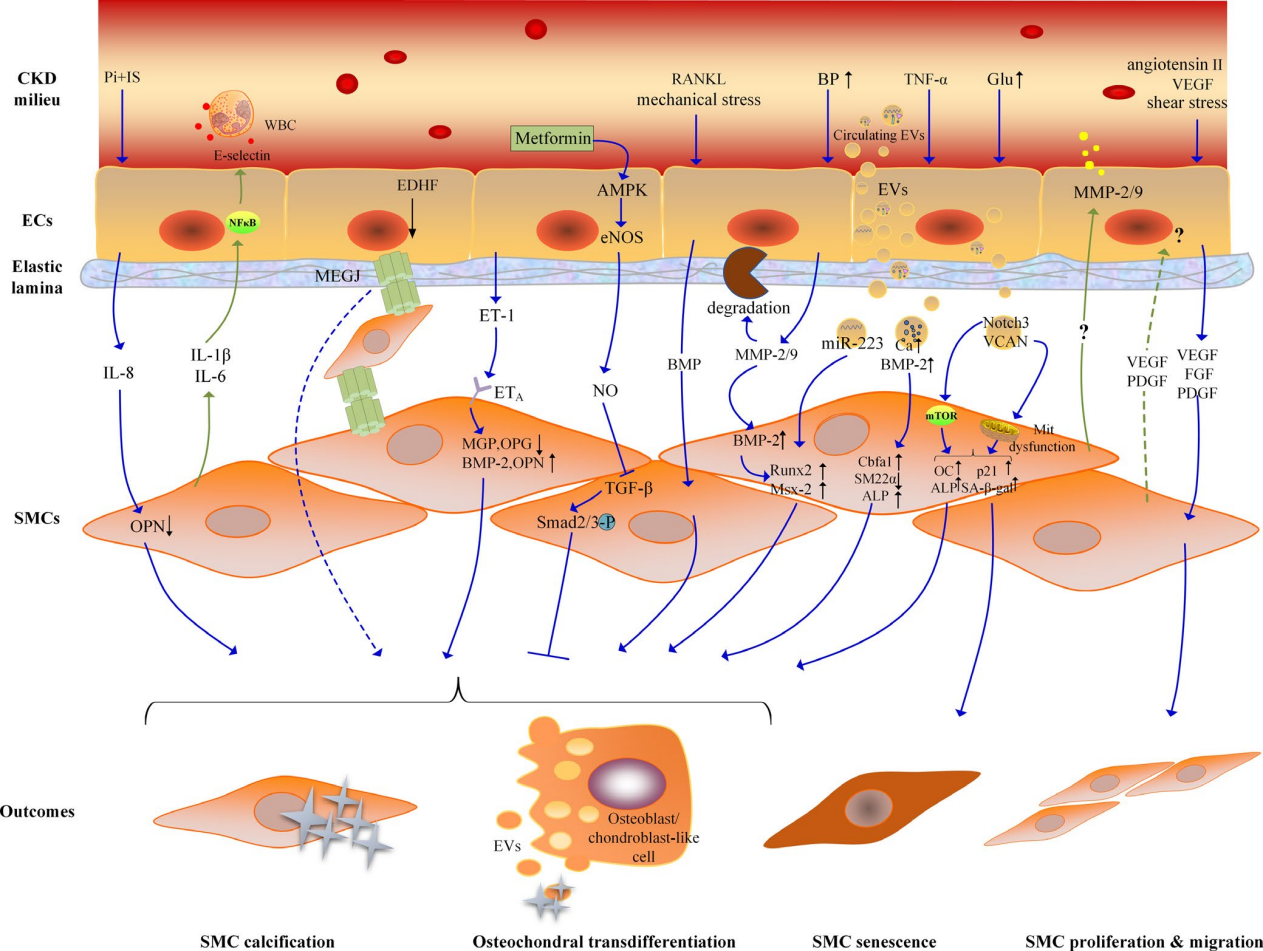
Crosstalk between ECs and SMCs in CKD‐associated VC. From top to bottom, there are schematic diagrams of the lumen, vascular endothelial layer, elastic lamina, vascular smooth muscle cell layer and the outcomes of SMCs. The CKD milieu means a variety of stimulating factors with high levels of inflammation, oxidative stress, glucose, PTH, mechanical stress, etc. ECs and SMCs interact with each other through inflammation, MEGJ, vasodilator (eg NO), vasoconstrictor (eg ET‐1), pro‐calcification factor (eg BMP), MMPs, EVs and pro‐angiogenic and pro‐fibrotic growth factors (eg VEGF), etc. The blue arrowed line represents the effect of ECs on SMCs and the green arrowed line represents the effect of SMCs on ECs. Abbreviation: SMC, smooth muscle cell; EC, endothelial cell; IL, Interleukin; TNF‐α, tumor necrosis factor α; TGF‐β, transforming growth factor β; Pi, phosphate; IS, indoxyl sulphate; WBC, white blood cell; BP, blood pressure; Glu, glucose; RANKL, receptor activator of NFκ‐β ligand; OPN, osteopontin; MGP, matrix Gla protein; OPG, osteoprotegerin; BMP, bone morphogenetic protein; ET‐1, endothelin‐1; MEGJ, myoendothelial gap junctions; NO, nitric oxide; eNOS, endothelial nitric oxide synthase; EDHF, endothelial‐derived hyperpolarizing factor; EVs, extracellular vesicles; MMP, matrix metalloproteinase; Runx2, runt‑related transcription factor2; MSX2, muscle segment homeobox 2; Cbfα1, core binding factor alpha 1; ALP, alkaline phosphatase; OC, osteocalcin; SA‑β‑gal, senescence‑associated β‑galactosidase; Mit dysfunction, mitochondrial dysfunction; VEGF, vascular endothelial growth factor; FGF, fibroblast growth factor; PDGF, platelet‐derived growth factor

VC is a process mediated by multiple cell types, but our understanding of it is far from sufficient. SMCs seem to play a central role in VC. However, their physiological functions greatly depend on normal EC behaviour, and their pathological changes also require regulation by ECs since ECs are the main sensors of circulating pathological triggers. Communication through multiple biomolecules and signalling pathways between these two kinds of cells does have important significance, especially in VC associated with CKD (Figure 1).

### 5.1 ECs assist SMCs in promoting VC in CKD

#### 5.1.1 Inflammation‐mediated effects on VSMCs produced by ECs in CKD‐related VC

Under pro‐calcification factor stimulation, ECs and SMCs can release various pro‐inflammatory mediators, such as TNF‐α, ILs and ROS, to interact with each other. In particular, in patients with CKD, uraemic toxins, such as inorganic Pi and IS, cause osteogenic differentiation of VSMCs. These toxins can also change the function of ECs. In vitro studies have confirmed that Pi and IS can induce ECs to secrete IL‐8, which exacerbates the calcification of VSMCs. The main underlying mechanism may be preventing the production of OPN rather than inducing the osteogenic differentiation of SMCs. Blocking IL‐8 receptors or silencing the expression of IL‐8 genes in ECs can remarkably weaken the pro‐calcification effect of ECs.^49^ In addition, studies have confirmed that SMCs can undergo not only calcification but also osteogenic transdifferentiation, senescence, apoptosis and migration under stimulation by TNF‐α, ILs and other pro‐inflammatory cytokines.^19^, ^50^, ^51^ ECs, as ‘endocrine organs’, can release these pro‐inflammatory factors and may mediate diverse processes of SMCs to promote VC.

#### 5.1.2 Pro‐angiogenic and pro‐fibrotic growth factors secreted by ECs induce SMC proliferation and migration to promote VC in CKD

Exposure to the uraemic milieu damages ECs, causing the release of not only pro‐inflammatory cytokines but also pro‐angiogenic growth factors and pro‐fibrotic growth factors, such as vascular endothelial growth factor (VEGF), fibroblast growth factor (FGF) and platelet‐derived growth factor (PDGF), which exacerbate EC injury and potentially stimulate VSMC proliferation, migration and dedifferentiation to promote VC.^51^, ^52^ In particular, SMC proliferation and migration contribute to the development of arterial stiffness and atherosclerosis, both of which are preludes to VC. In vitro, ECs injured by angiotensin II can produce a surfeit of VEGF, leading to the proliferation and migration of SMCs co‐cultured in a non‐contact system with ECs.^53^ Moreover, VEGF‐treated ECs can secrete increased amounts of FGF2, thereby stimulating the proliferation and migration of SMCs, which also means that VEGF can affect SMCs in an indirect manner.^54^ Animal experiments confirmed that enhancement of the FGF signalling pathway in ECs may exacerbate atherosclerosis. The following are some of the reasons involved. ECs overexpressing the FGF receptor exhibit increased apoptosis and expression of adhesion molecules, which can recruit inflammatory cells as well as SMCs and PDGF. The proliferation of SMCs in the aorta of EC‐targeted FGF receptor‐overexpressing mice is twice as high as that in control mice, which is thought to be caused by PDGF secreted by ECs.^55^ At the same time, low shear stress can also induce ECs to secrete PDGF‐BB, promoting VSMC migration and proliferation in a paracrine manner.^52^ Pro‐angiogenic growth factors and pro‐fibrotic growth factors perform vital roles in vascular remodelling and may also be involved in CKD‐related VC, a type of vascular remodelling.

#### 5.1.3 ECs affect VSMCs via MMPs in CKD‐related VC

MMPs are important not only in elastin degradation but also in guiding the phenotypic transformation and communication of cells in VC. The expression and activity of MMP‐2/9 tend to increase in CKD patients with cardiovascular disease.^56^ Additionally, VSMCs from CKD rats have upregulated expression of MMP‐2/9, which are closely related to VC. Blockade of MMP activity can inhibit arterial calcification.^57^ Co‐culture and culture with conditioned medium in vitro have confirmed that ECs can increase the deposition of calcium, the activity of ALP and the expression of Runx2, Msx2, BMP2 and osterix in SMCs isolated from spontaneously hypertensive rats by secreting soluble factors. It is believed that elevated expression of MMP‐2 and MMP‐9 in ECs may be a mechanism for promoting SMC calcification.^58^ Gelatinases promote the phenotypic conversion of VSMCs, which manifests as the expression of Runx2 and Msx2, by upregulating BMP2 expression.^59^ These data suggest significant roles for MMPs in VSMC phenotypic transformation, matrix re‐establishment and VC. Notably, MMPs can serve to mediate the communication between ECs and SMCs in CKD‐related VC.

#### 5.1.4 A pro‐calcification factor (BMP) from ECs induces osteochondral differentiation in SMCs in VC

BMP, a member of the transforming growth factor (TGF)‐β superfamily, is acknowledged to be a key regulator of bone development and vascular calcification. As a pro‐calcification factor, it is known for its role in inducing the osteochondral transdifferentiation of ECs and SMCs. Receptor activator of NFκ‐β ligand (RANKL) can induce HAECs to release BMP2 and promote VC. Interestingly, RANKL induces the release of BMP2 by HAECs rather than by human aortic smooth muscle cells (HASMCs) and cannot trigger the osteogenic activities of HASMCs directly. Instead, BMP2 is secreted by ECs to promote the osteogenic activities of HASMCs. This phenomenon indicates the importance of BMP‐mediated crosstalk between ECs and SMCs in VC.^60^ Mechanical stress activates ECs and upregulates the expression of BMP2, which further regulates the expression of osteogenic genes in SMCs.^45^ MMPs, as mentioned above, may also participate in the interaction through BMP signalling.^59^ BMPs can also be loaded into EVs to participate in crosstalk between cells. The related content of EVs will be explained in detail later. Overall, the pro‐calcification factor BMP participates in the interaction between ECs and SMCs in VC.

#### 5.1.5 EV‐mediated communication between ECs and SMCs in CKD‐related VC

EVs are encapsulated by a lipid bilayer membrane, which is interspersed with bioactive ligands and selectively loaded with cargo, including various RNA transcripts, DNA fragments, lipids, cytokines and proteins. Released by most types of cells, EVs can mediate cell‐to‐cell communication by delivering distinct cargo from the sender cell to the recipient cell, and bioactive ligands on the membrane surface may also convey information.^61^ In CKD patients, circulating EVs loaded with low fetuin‐A and GRP (Gla‐Rich Protein) can be actively taken up by VSMCs, thus inducing VSMC osteochondrogenic transdifferentiation and inflammatory processes to promote VC.^62^ Interestingly, Cavallari et al^63^ found that among the majority of circulating EVs in haemodialysis patients, such as those derived from platelets, monocytes/macrophages and ECs, only endothelial EVs showed a significant increase in the patients compared with healthy subjects, which suggested that circulating EVs in CKD patients might mainly be driven by ECs. These EVs can promote VSMC calcification in vitro and cause endothelial dysfunction, such as increased human umbilical vein endothelial cell (HUVEC) apoptosis and reduced angiogenesis. The mechanism is related to the upregulation of the EV cargo‐miR‐223 level.^63^ Another in vitro experiment also confirmed that under the induction of uraemic toxin IS, injured ECs can produce excessive microvesicles, which mediate calcium deposition, inflammation and osteogenic transdifferentiation of SMCs.^64^

CKD is closely related to inflammation, hyperglycaemia, etc. It has been found that under stimulation with the inflammatory mediator TNF‐α, ECs can secrete BMP‐2 and endothelial microparticles (EMPs) with high calcium and BMP‐2 concentrations and induce osteogenic differentiation and calcification in VSMCs, which are similar to the effects produced with EMPs obtained from patients with CKD^65^ and senescence‐related ECs.^65^, ^66^ Under high‐glucose stimulation, HUVECs can induce calcification and senescence in VSMCs, which manifest as increased OC and p21 expression levels and simultaneously increased mineralized nodules and senescence‑associated β‑galactosidase (SA‑β‑gal)‐positive cell numbers. Exosomes loaded with Notch3 communicate between two kinds of cells, and the mTOR signalling pathway is involved.^67^ A team also found that HUVEC‐derived exosomes exposed to high‐glucose concentrations contained elevated levels of versican (also known as VCAN, a chondroitin sulphate proteoglycan proven to appear in the mitochondria of VSMCs), were taken up by recipient VSMCs and induced mitochondrial dysfunction, resulting in oxidative stress, calcification and senescence in VSMCs.^68^ Notably, in a positive sense, EMPs triggered by EC apoptosis can reduce the proliferation and migration of VSMCs and subsequent neointima formation with the transfer of cargo‐miR126 to VSMCs.^69^

Overall, EVs participate in cell‐to‐cell communication by conveying miRNAs, pro‐calcification proteins, increased levels of calcium and phosphorus metabolites, etc. and regulate cell phenotypic transformation or serve as nucleation sites for calcification. EVs may also help form calcified nodules and directly calcify the extracellular matrix. EVs, as cell communicators, may be targets for blocking harmful processes, tools for delivery of beneficial substances and markers of diagnosis in future treatments. In this regard, as an emerging research field, EV‐mediated intercellular communication promoting VC in CKD remains to be explored.

#### 5.1.6 Vasodilator‐, vasoconstrictor‐ and myoendothelial gap junction (MEGJ)‐mediated interactions between ECs and SMCs in CKD‐related VC

The interaction between ECs and SMCs is widely recognized to be involved in regulating vessel tone. Vascular stiffness is closely related to VC, especially medial calcification, both of which are attributable to various pathological states of CKD.^70^, ^71^ It is known that ECs stimulated with mechanical stress, vasoactive molecules, etc. can affect SMCs and regulate vessel tone through vasodilators (eg NO) and vasoconstrictors (eg endothelin‐1 (ET‐1)). Of interest, current evidence suggests that NO and ET‐1 may be involved in regulating VC. In vitro, NO can prevent the calcification and osteochondrogenic transdifferentiation of SMCs by inhibiting TGFβ‐induced Smad2/3 phosphorylation.^72^ eNOS is closely related to NO production. Metformin can attenuate VC via the AMPK/eNOS/TGF‐β1 signalling pathways in rats.^73^ These findings suggest an important role of NO in VC. ET‐1 is also involved in VC by inhibiting calcification inhibitors, such as MGP and osteoprotegerin (OPG), while increasing the levels of pro‐calcification factors, such as BMP2 and OPN.^74^ However, VC can be reduced by antagonists of the ET‐1 A receptor located in SMCs in CKD rats.^75^

In addition, ECs can regulate VSMC relaxation though endothelial‐derived hyperpolarizing factors (EDHFs), which act on MEGJs. MEGJs, which are located in the internal elastic lamina, couple ECs and SMCs in physical, electrical and metabolic manners, which makes the interaction direct.^76^ Many studies have found that multiple signalling pathways in ECs and SMCs can be mediated by MEGJs, which play roles in different kinds of vascular dysfunction. It has been found that serotonin secreted by pulmonary artery ECs enters pulmonary artery SMCs through MEGJs and then induces TGF‐β signalling and differentiation in SMCs.^77^ Blocking communication mediated by MEGJs prevents TGF‐β signalling and differentiation of pulmonary artery SMCs.^78^ These studies suggest that the transit of regulatory molecules though MEGJs may serve as an alternative to the paracrine pathway between ECs and SMCs and emphasize the important role for MEGJ‐mediated direct communication in ECs and SMCs. This process was gradually discovered in atherosclerotic lesions.^79^, ^80^ In particular, shear stress acting on HCAECs causes the dysfunction of MEGJs, which induces human coronary aortic smooth muscle cell (HCASMC) transition to the ‘synthetic phenotype’ associated with atherosclerosis.^80^ Since atherosclerosis often results in intimal calcification, MEGJ‐mediated direct crosstalk between ECs and SMCs may also play an important role in VC. In conclusion, the roles of MEGJs in VC may mainly involve regulating vessel tone and transmitting information, which are areas worthy of exploration in CKD‐related VC. Thus, vasodilators, vasoconstrictors and MEGJs may be involved in the crosstalk between ECs and SMCs in VC.

### 5.2 SMCs act on ECs, creating a crosstalk loop that further promotes VC

The crosstalk between ECs and SMCs in VC is also reflected in the role of SMCs in relation to ECs. On the one hand, SMCs may directly act on another ‘protagonist’ in VC—ECs. On the other hand, SMCs provide a loop via feedback, which amplifies the effect of ECs on SMCs. As mentioned earlier, pro‐inflammatory cytokines can induce injury and osteogenic transdifferentiation in both ECs and SMCs. Inflammation may mediate not only the effect of ECs on SMCs but also that of SMCs on ECs. Synthetic smooth muscle cells (sSMCs), which are transformed from a ‘contractile phenotype’ under pathological states, can induce inflammatory signalling and gene expression in ECs in turn. The pro‐inflammatory cytokines IL‐1β and IL‐6 synthetized by sSMCs induce ECs to secrete the adhesion molecule E‐selectin.^81^ E‐selectin can mediate adhesion between ECs and inflammatory cells, which may result in further release of inflammatory factors acting on ECs and SMCs to aggravate VC. In an inflammatory environment, TNF‐α‐activated ECs can induce VSMCs to express the pro‐inflammatory cytokines TNF‐α, IL‐6 and VEGF.^82^ These pro‐inflammatory cytokines may also act on ECs, promoting their osteogenic activities and simultaneously strengthening their effects on SMCs.

Additionally, in in vitro studies, SMCs co‐cultured with ECs expressed more VEGF, PDGF and TGF‐β than those cultured without ECs. Compared with ECs cultured alone, ECs co‐cultured with VSMCs synthesized a higher level of VEGF.^83^ These results suggest that SMCs and ECs affect each other in some way. VEGF, PDGF and FGF are known to induce proliferation and migration in SMCs and ECs, promoting atherosclerotic progression and vascular stiffness, which are precursors to VC. Low shear stress, which plays an important role in the calcification of atherosclerotic lesions, ultimately increases the migration and proliferation of ECs and VSMCs when applied to ECs. ECs can not only affect VSMCs by secreting PDGF‐BB but also undergo regulation by VSMCs as a positive feedback response to their control of SMCs via TGF‐β1.^52^ In addition, SMCs conversely provide feedback to ECs to regulate their proliferation and migration, which may also be involved in VC.

In CKD‐related VC, BMP expression is usually upregulated during the osteogenic activities of SMCs. BMP also plays a vital role in the osteogenic transdifferentiation of ECs. SMCs may also affect ECs by releasing BMP, similar to how ECs affect SMCs. Notably, it was also found that in ECs co‐cultured with SMCs, MMP‐2 and MMP‐9 levels were increased compared with those in ECs cultured without SMCs, which demonstrated that the crosstalk between ECs and SMCs affects both cell types. Here, the crosstalk intensified calcification as well.^58^ Although some data show conflicting results, the FGF23/αKlotho axis is believed to be linked to uraemic vasculopathy. Hu MC et al. proposed the concept of the ‘endothelial‐vascular smooth muscle complex’ and considered it to be a potential target of αKlotho in VC in the context of CKD, emphasizing crosstalk between the two kinds of cells.^51^ These studies revealed the effects of SMCs on ECs, and the communication between ECs and SMCs may play a crucial role in CKD‐related VC. However, research data in this area are still severely lacking. In particular, studying the effects of SMCs on ECs to further elucidate the crosstalk loop is necessary.

## 6 CONCLUSION

In this review, for the first time, we have explained in more detail the roles of ECs and the crosstalk between ECs and SMCs in CKD‐related VC. However, what researchers have found thus far is just the ‘tip of the iceberg’ in this complex network and is far from sufficient. In the future, it is important and significant to further explore this interesting area. As more clues are discovered, comprehensive therapies targeting endothelial layers and cellular interaction networks will provide clinicians with new insights into the prevention and treatment of VC and reduce the incidence of cardiovascular events in CKD.

## CONFLICT OF INTEREST

The authors declare that there are no conflicts of interest.

## AUTHOR CONTRIBUTIONS

YXZ drafted the manuscript; YXZ and LTW prepared the figure. RNT and BCL provided critical revisions. All authors revised and approved the final manuscript before submission.

## Data Availability

Data available on request.
